# Supplementary material for: Stacking Model‐Based Classifiers for Dealing With Multiple Sets of Noisy Labels
Source: Biom J. 2025 Mar 12;67(2):e70042. doi: 10.1002/bimj.70042 (PMC11898607; doi:10.1002/bimj.70042)
Supplement: Supplementary file 1 — Supporting Information [file BIMJ-67-e70042-s001.zip › density-based-ensemble-model-main/competitors_real_data/netlab-master/netlab-master/help/gtmpost.htm]

Netlab Reference Manual gtmpost


# gtmpost

## Purpose

Latent space responsibility for data in a GTM.

## Synopsis

```
post = gtmpost(net, data)
[post, a] = gtmpost(net, data)
```

## Description

`post = gtmpost(net, data)` takes a GTM
structure `net`, and computes the responsibility at each latent space
sample point `net.X`
for each data point in `data`.

`[post, a] = gtmpost(net, data)` also returns the activations
`a` of the GMM `net.gmmnet` as computed by `gmmpost`.

## See Also

`gtm`, `gtmem`, `gtmlmean`, `gmlmode`, `gmmprob`

---

**Pages:**
Index


---

Copyright (c) Ian T Nabney (1996-9)
